# Efficient Organic Light Emitting Diodes Using Solution-Processed Alkali Metal Carbonate Doped ZnO as Electron Injection Layer

**DOI:** 10.3389/fchem.2019.00226

**Published:** 2019-04-16

**Authors:** Guo Chen, Feiyang Liu, Zhitian Ling, Pengpeng Zhang, Bin Wei, Wenqing Zhu

**Affiliations:** Key Laboratory of Advanced Display and System Applications, Shanghai University, Ministry of Education, Shanghai, China

**Keywords:** organic light emitting diodes (OLED), solution process, electron injection layer, doped ZnO, alkali metal carbonate

## Abstract

In this study, we demonstrate highly efficient, inverted organic light-emitting diodes (IOLEDs) using solution-processed alkali metal carbonate doped ZnO as an electron injection layer (EIL) and tris-(8-hydroxyquinoline) aluminum (Alq_3_) as an emitter layer. In order to enhance the electron injection efficiency of the IOLEDs, the ZnO EIL layers were modified by doping various alkali metal carbonate materials, including Li_2_CO_3_, Na_2_CO_3_, K_2_CO_3_, and Cs_2_CO_3_, using the low-temperature wet-chemical method. Compared to the control neat ZnO EIL-based IOLEDs, the alkali metal carbonate doped ZnO EIL-based IOLEDs possess obviously improved device performance. An optimal current efficiency of 6.04 cd A^−1^ were realized from the K_2_CO_3_ doped ZnO EIL based IOLED, which is 54% improved compared to that of the neat ZnO EIL based device. The enhancement is ascribed to the increased electron mobility and reduced barrier height for more efficient electron injection. Our results indicate that alkali metal carbonate doped ZnO has promising potential for application in highly efficient solution-processed OLEDs.

## Introduction

Organic light-emitting diodes (OLEDs) have been extensively investigated as a promising technology for energy-saving lighting and large-area flexible displays (Kido et al., 1995; Forrest, 2004; Sasabe and Kido, 2010; Higuchi et al., 2015; Li et al., 2016). Typically, there are two kinds of device structures mainly used in OLEDs, i.e., conventional and inverted structures. Compared with the conventional structure, the inverted OLEDs (IOLEDs), which use the air stable metals as top anode and indium-tin-oxide (ITO) as the cathode, have been considered as an advantageous approach to improve the stability and the roll-to-roll fabrication process of flat-panel display (Chu et al., 2006; Morii et al., 2006; Sessolo and Bolink, 2011; Chen et al., 2012; Guo et al., 2017; Fukagawa et al., 2018). Moreover, one benefit of the IOLED is to take advantage of the existing n-type amorphous silicon thin film transistor (a-Si TFT) technology for the development of the active-matrix driving OLED technology (Kabra et al., 2010; Hsieh et al., 2011; Zhong et al., 2011; Park et al., 2015; Hosono et al., 2017).

In an IOLED device, ITO is used as the electron injection cathode while metal electrode acts as hole injection anode. To facilitate electron injection from ITO into the upper electron transporting or light-emitting layers, the electron injection layers (EILs) are usually coated onto the ITO cathode. Moreover, introducing the EIL into the OLED devices can also efficiently reduce the driving voltage and improve the power efficiency in the same time (Guo et al., 2017). Up to date, some inorganic alkali metal-containing materials (such as LiF and Cs_2_CO_3_), metal oxide materials (such as TiO_2_, ZnO, and ZrO_2_) and ionic π-conjugated polymers have been reported as efficient EIL for high performance OLEDs (Tokmoldin et al., 2009; Kim et al., 2014; Chiba et al., 2015; Zhao et al., 2017). Among all these EIL materials, ZnO has attracted increasing attention for being used in IOLED because ZnO is environmentally stable, low-cost and has high transparency in visible region (Chen et al., 2016). In particular, the solution-processed ZnO film has been widely used as an EIL in the IOLED configuration due to its advantages of optical transparency, low work function, high electron mobility and good electron selective, and hole blocking contact (Bolink et al., 2007; Chiba et al., 2012; Dong et al., 2017). However, the ZnO EIL based IOLED demonstrates ordinary device performance owing to the injection barrier between ZnO EIL and the adjacent electron transporting layers (ETL) caused by the still high work function of ZnO film (Höfle et al., 2014). Thus, there is an urgent need to develop a new technique to improve the injection efficiency of ZnO EIL based IOLEDs.

Some recent reports indicate that doping the low-concentration group I elements into the ZnO host is one of the most efficient methods to enhance the optoelectronic properties of ZnO films for organic electronic devices (Lin et al., 2016; Nho et al., 2016; Wang et al., 2018). The doped ZnO films always demonstrate much higher electron mobility and reduced work function than the bare ZnO film, which are sought-after properties for high efficiency organic electronic devices. For example, the Li ion and LiF have been doped in ZnO films to increase the electron mobility and thus increase the charge collection efficiency and reduce the charge carrier recombination, leading to enhanced photovoltaic performance in organic solar cells (Chang et al., 2013; Lin et al., 2016). To obtain the doped ZnO film, various techniques such as sputting, pulsed laser deposition, sol-gel, chemical vapor deposition, thermal evaporation, spray pyrolysis, and wet-chemical method have been used to dope the dopant into the ZnO host (Chen et al., 2016). Among these doping techniques, wet-chemical doping is the most promising route due to its advantages of low-cost, easy operation and low-temperature (Chang et al., 2015). It has been well used in the research field of organic solar cells and thin film transistors (Chang et al., 2013, 2015). However, to the best of our knowledge, few researchers pay attention to the usage of wet-chemical doping technique to fabricate doped ZnO films as EILs for OLEDs.

In this work, to systemically investigate the effect of group I elements doping on properties of the ZnO films and the doped ZnO film based IOLEDs, we introduced various alkali metal carbonates (M_2_CO_3_, M = Li, Na, K, and Cs) with various doping ratios into ZnO film using the low-temperature wet-chemical doping method. Then the doped ZnO:M_2_CO_3_ films were employed as EIL layers, combined with the tris-(8-hydroxyquinoline) aluminum (Alq_3_) emitter layer, to construct the IOLEDs. By controlling the doping concentration of M_2_CO_3_ in the precursor solution, highly efficient IOLEDs with high current efficiency and power efficiency have been obtained.

## Experimental Section

### Materials

All materials employed in this study were purchased from commercial sources and used without further purification. The ZnO precursor solution was prepared by dissolving ZnO powder (Sigma Aldrich, 99.9%) in an ammonium solution (Sigma Aldrich, 99%) to form a 0.1 M $\text{Zn}{\left({\text{NH}}_{3}\right)}_{4}^{2+}$ complex precursor saturated solution. The solution was refrigerated for several tens of hours to promote the ZnO powder dissolution. Then, various molar ratios (3, 5, and 10%) of M_2_CO_3_ were added to the precursor solution to prepare the M_2_CO_3_ doped $\text{Zn}{\left({\text{NH}}_{3}\right)}_{4}^{2+}$ complex precursor solution. The patterned indium-tin-oxide (ITO) glass substrates with a sheet resistance of 15 Ω/sq were cleaned in ultrasonic bath with detergent, de-ionized water, acetone, and isopropanol consecutively, then they were dried by keeping them in an oven at 80°C for 12 h before usage.

### Film Characterization

The UV-Vis transmittance was recorded at room temperature using an ultraviolet-visible-near infrared spectrophotometer (U-3900H, Hitachi). The work function was determined using a Riken-Keiki AC-3 ultraviolet photoelectron spectrometer. X-ray photoelectron spectroscopy (XPS) measurements were implemented using X-ray photoelectron spectrometer (Kratos Amicus Budget). The surface morphology of the film was investigated by the atomic force microscopy (AFM) technique in contact mode using the Seiko instrument SPA 400 AFM system. The films for the above mentioned measurements were prepared under the same conditions used for fabricating the devices to enable accurate comparisons.

### Device Fabrication And Characterization

**Figure 1:**
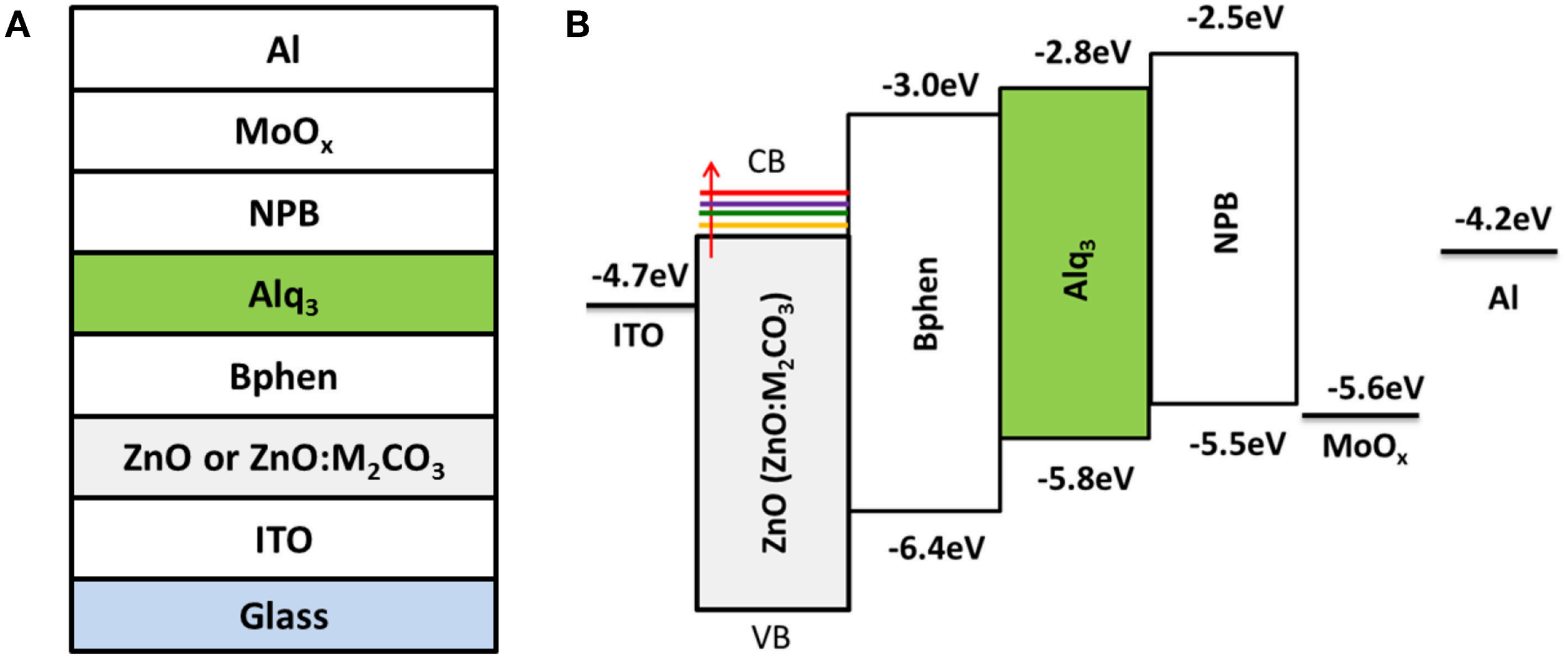
**(A)** Device structure of the alkali metal carbonate doped ZnO (ZnO:M_2_CO_3_) EIL based inverted organic light-emitting diodes (IOLEDs) and **(B)** the energy-level diagram of the materials under investigation.

The IOLEDs were fabricated with a structure of ITO/ZnO:M_2_CO_3_(10 nm) /4,7-diphenyl-1,10-phenanthroline(Bphen, 20 nm)/Alq_3_(20 nm)/N,N′-bis(naphthalen-1-yl)-N,N′-bis(phenyl)-benzidine (NPB, 40 nm)/MoO_x_(5 nm)/Al (120 nm), as shown in Figure 1A. Firstly, the clean ITO substrates were treated by ultraviolet-ozone for 30 min. Then the ZnO:M_2_CO_3_ films were prepared by spin-coating the $\text{Zn}{\left({\text{NH}}_{3}\right)}_{4}^{2+}$:M_2_CO_3_ precursors on the ITO substrates and subsequently heated at 200°C for 15 min to realize the conversion from $\text{Zn}{\left({\text{NH}}_{3}\right)}_{4}^{2+}$:M_2_CO_3_ complex to ZnO:M_2_CO_3_ (Chang et al., 2015). Then the substrate was transferred into a high-vacuum chamber (1 × 10^−5^ Pa), where organic layers and a metal cathode layer were successively evaporated using a shadow mask. The deposition rates for the organic materials, MoO_3_ and Al, were 1.0, 0.5, and 5.0 A s^−1^, respectively. The electron-only devices were fabricated with a structure of ITO/ZnO:M_2_CO_3_ (10 nm)/Bphen (60 nm)/Liq (1 nm)/Al (120 nm). The device active area was 0.04 cm^2^, defined by the overlap of anode and cathode. The current density–voltage–luminescence (*J–V–L*) characteristics were measured using a Keithley 2400 source meter and a PR-650 Spectra Colorimeter. The luminance and spectra of each device were measured in the direction perpendicular to the substrate. All the measurements were performed in air under ambient conditions without device encapsulation.

## Results and Discussion

Figure 1 illustrates the device architecture of ZnO (ZnO:M_2_CO_3_) based IOLEDs and the corresponding energy-level diagram. In this IOLED device, the Alq_3_ film acts as an emitter layer, the NPB and Bphen films are hole-transporting and ETLs, respectively. Additionally, MoO_3_ and ZnO (ZnO:M_2_CO_3_) are employed as hole-injection layer and EIL, respectively. For comparison, all devices have the same emitter layer and functional layers, except that of the ZnO:M_2_CO_3_ layers. Various M_2_CO_3_ dopant, including Li_2_CO_3_, Na_2_CO_3_, K_2_CO_3_, and Cs_2_CO_3_ with various concentration, were doped into the ZnO film to investigate the effect of M_2_CO_3_ doping on the film properties of the ZnO EIL and the device performance of doped ZnO based IOLEDs.

**Figure 2:**
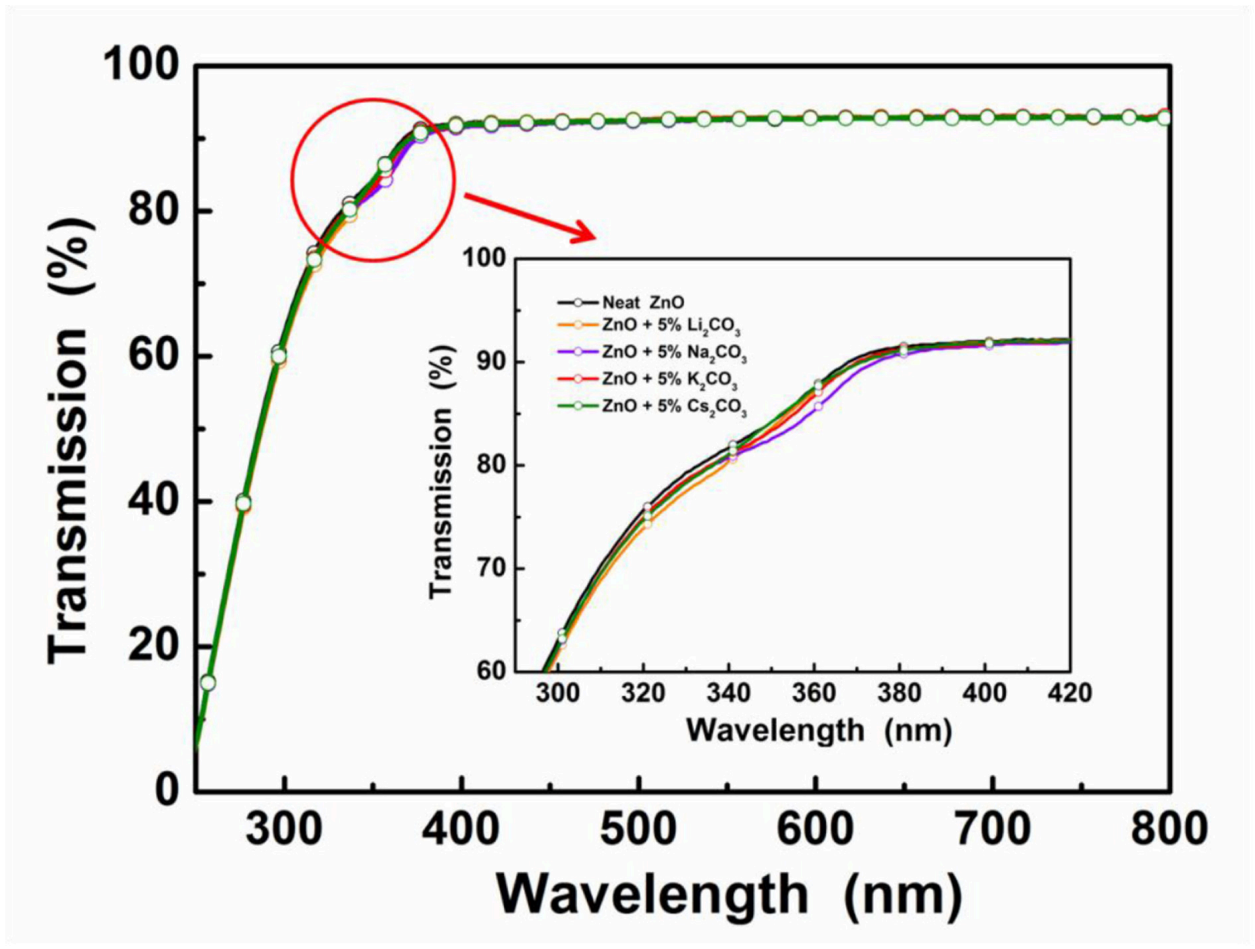
Transmittance spectra of the neat ZnO film and the doped ZnO:M_2_CO_3_ films coated on quartz substrates.

**Figure 3:**
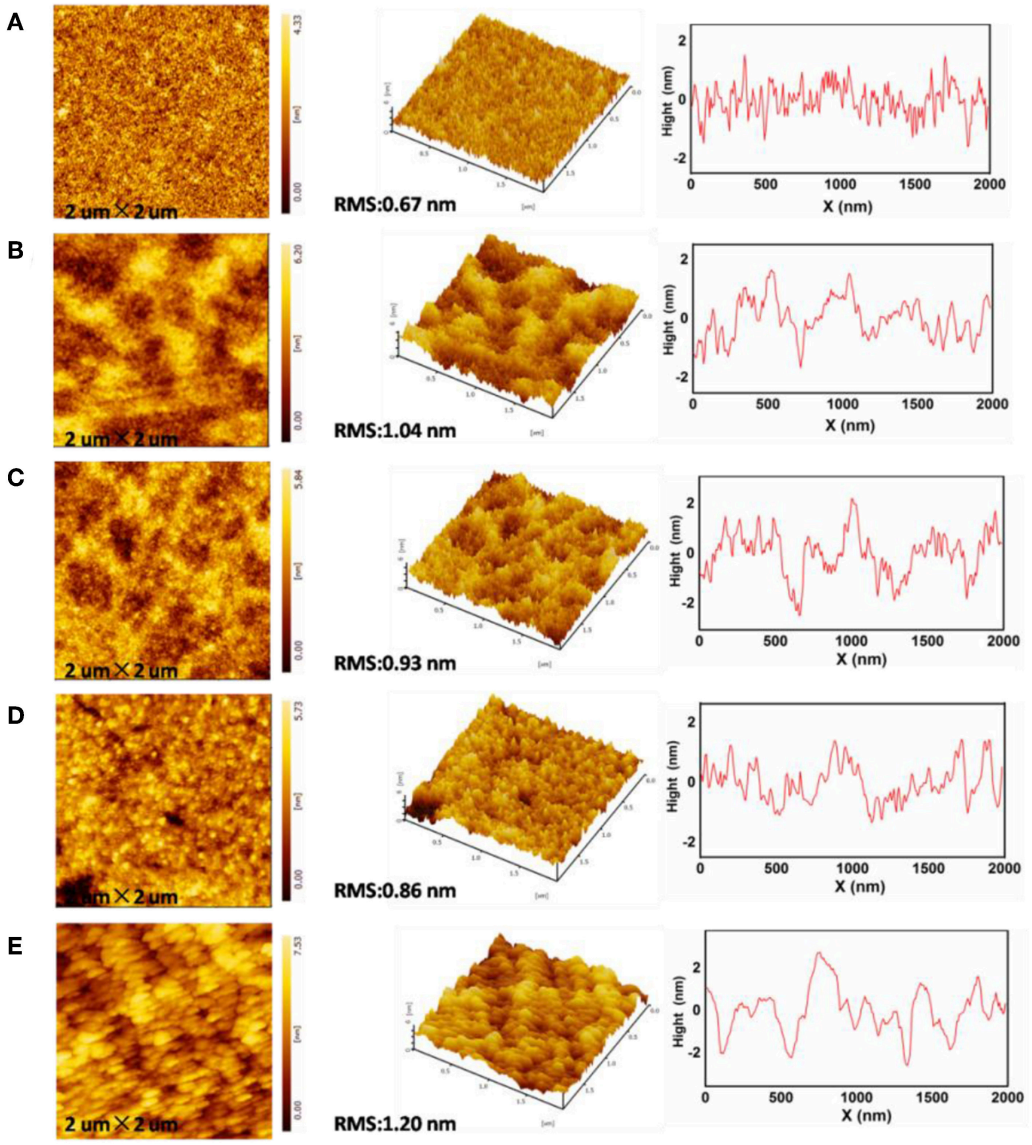
Atomic force microscopy (AFM) topographic and 3D images of **(A)** neat ZnO film, **(B)** ZnO:Li_2_CO_3_ film, **(C)** ZnO:Na_2_CO_3_ film, **(D)** ZnO:K_2_CO_3_ film, **(E)** ZnO:Cs_2_CO_3_ film.

**Figure 4:**
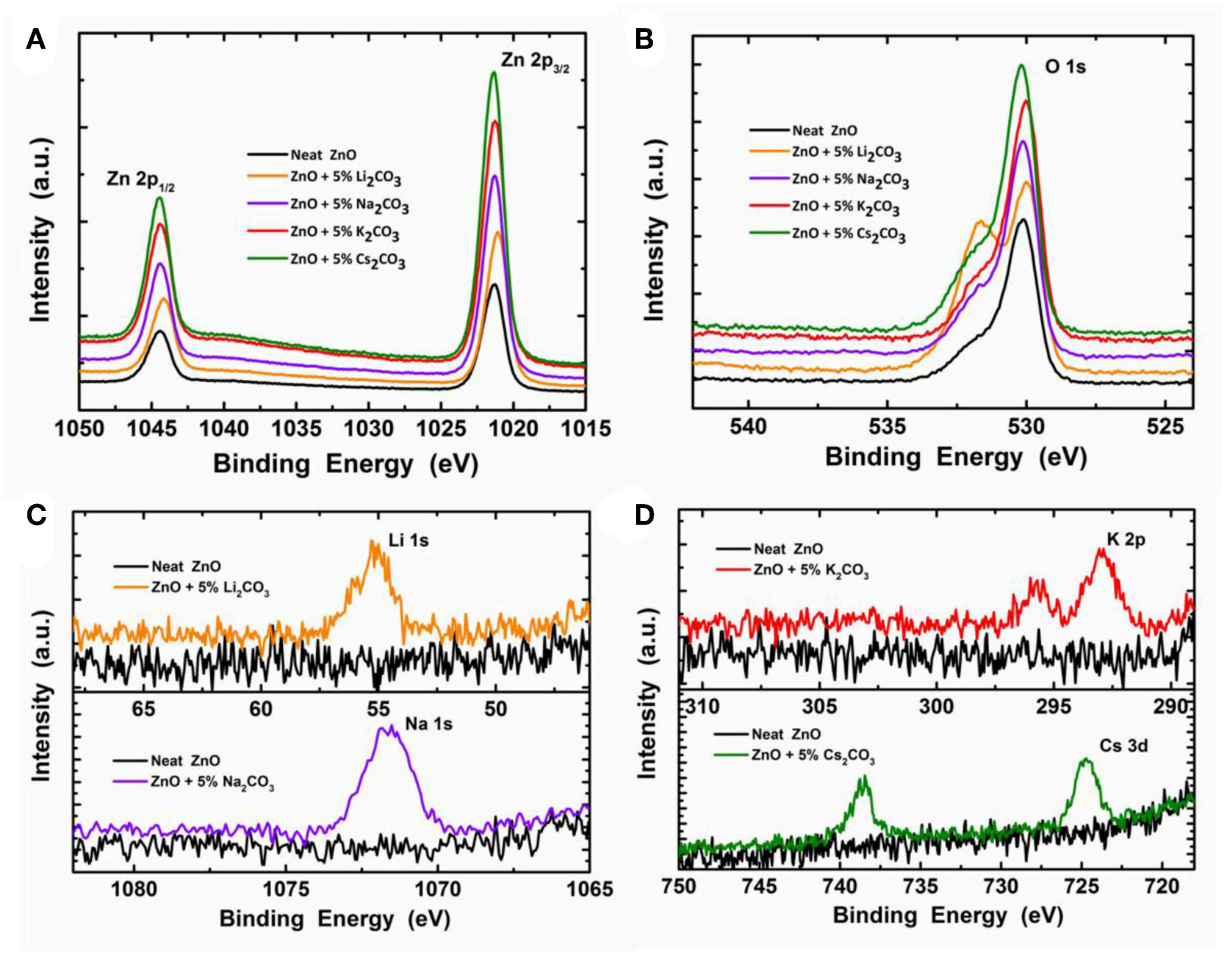
The X-ray photoelectron spectroscopy (XPS) spectra of the neat ZnO film and ZnO:M_2_CO_3_ doped films coated on Si wafer: **(A)** Zn 2p_1/2_ and Zn 2p_3/2_; **(B)** O 1s; **(C)** Li 1s, Na 1s, and **(D)** K 2p and Cs 3d.

**Figure 5:**
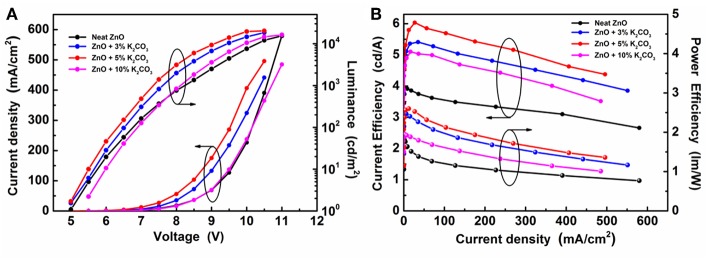
**(A)** The J-V-L characteristics and **(B)** the current efficiency and power efficiency characteristics of IOLEDs based on the neat ZnO and the doped ZnO:K_2_CO_3_ EILs with various K_2_CO_3_ doping concentration.

**Figure 6:**
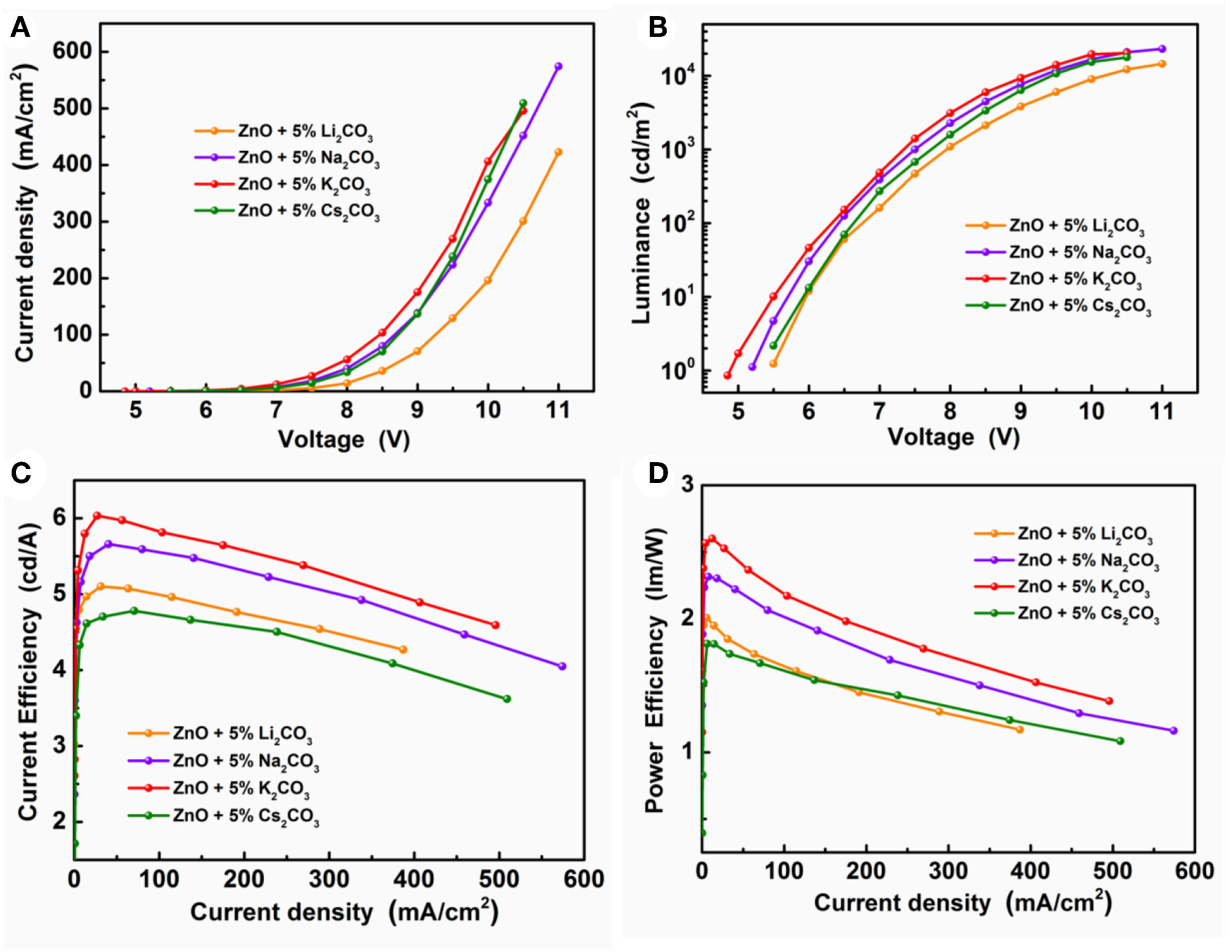
**(A)** The J-V characteristics, **(B)** the L-V characteristics, **(C)** the current efficiency and **(D)** the power efficiency characteristics of the IOLEDs based on the doped ZnO:Li_2_CO_3_, ZnO:Na_2_CO_3_, ZnO:K_2_CO_3_, and ZnO:Cs_2_CO_3_ EILs.

**Figure 7:**
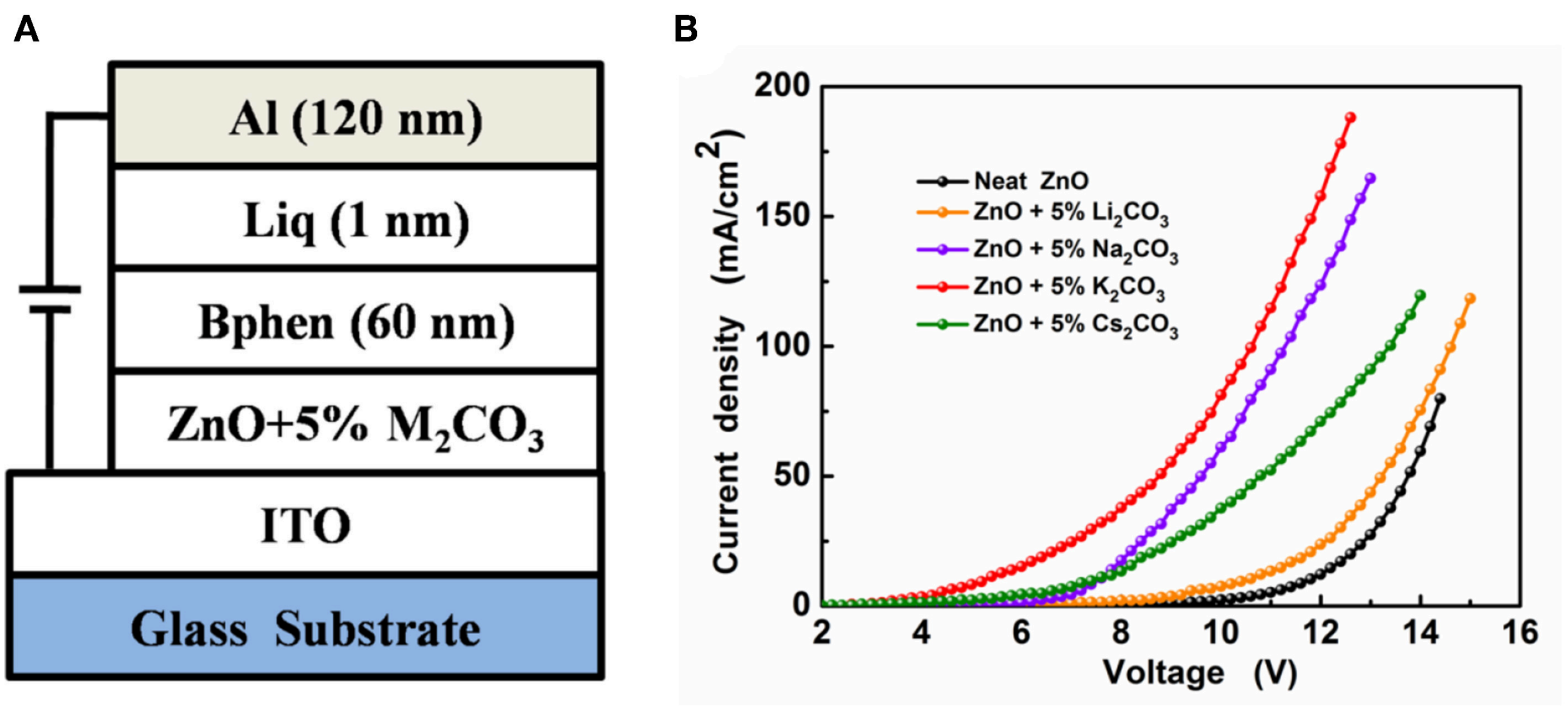
**(A)** The device structure of the electron-only devices and **(B)** the J-V characteristics of the electron-only devices.

The ZnO:M_2_CO_3_ EILs in this study were fabricated by a simple wet-chemical doping method using the blend aqueous solution of M_2_CO_3_ and Zn-ammine complex as the precursor. The detailed fabrication process includes the following three steps: (1) The $\text{Zn}{\left({\text{NH}}_{3}\right)}_{4}^{2+}$ complex precursor was prepared by dissolving ZnO powder in an ammonium solution (Lin et al., 2014). Then various M_2_CO_3_ aqueous solution with various molar concentration were blended into the $\text{Zn}{\left({\text{NH}}_{3}\right)}_{4}^{2+}$ complex aqueous solution to prepare the $\text{Zn}{\left({\text{NH}}_{3}\right)}_{4}^{2+}$:M_2_CO_3_ precursors for the doped films; (2) The doped ZnO:M_2_CO_3_ films were fabricated by spin-coating the $\text{Zn}{\left({\text{NH}}_{3}\right)}_{4}^{2+}$:M_2_CO_3_ precursors, followed by thermal annealing at 200°C for 15 min to realize the conversion from $\text{Zn}{\left({\text{NH}}_{3}\right)}_{4}^{2+}$ complex to ZnO. The fabrication route using the aqueous-based Zn-ammine complex solutions is one of the most promising routes to prepare the ZnO film, owing to its advantages of low-temperature and easy-operation. However, it is difficult to directly dope the metal ion into the ZnO host from the Zn-ammine complex precursor because the ammonium hydroxide tends to precipitate metal salts due to acid–base neutralization reaction (Zhang et al., 2018). Therefore, we choose the M_2_CO_3_ aqueous solutions with weak alkalinity to blend into the Zn-ammine complex aqueous solution to make the precursors for the doped ZnO:M_2_CO_3_ films. Herein, the weak alkalinity of the M_2_CO_3_ aqueous solution makes it coexist with the Zn-ammine complex. The doping concentration was controlled by blending various amounts of M_2_CO_3_ into the Zn-ammine complex aqueous solution. As shown in Figures 2–**7**, Figures S1–S3, Tables 1, 2 and Table S1, to investigate the effect of alkali metal doping on properties of the ZnO film and doped ZnO film based IOLED devices, we used various tools including UV-vis absorption spectra, AFM, XPS, and AC-3 to characterize the surface morphology and optoelectronic properties of the doped ZnO film, then fabricated and characterized a series of ZnO:M_2_CO_3_ EILs based IOLEDs.

**Table 1:** Device performance of IOLEDs based on the neat ZnO or doped ZnO:K_2_CO_3_ EILs with various K_2_CO_3_ doping concentration.

**K_**2**_CO_**3**_ doping concentration**	**V_on_^a^ (V)**	**Luminance^**b**^ (cd/m^**2**^)**	**CE^**b**^ (cd/A)**	**PE^**b**^ (lm/W)**
0	5.2	15,430	3.92	1.81
3 wt%	4.9	20,500	5.41	2.46
5 wt%	4.8	22,651	6.04	2.61
10 wt%	5.5	16,500	5.01	1.90

a *The driving voltage for the luminance of 1 cd/m^2^*.

b *The maximum values*.

**Table 2:** Device performance of the IOLEDs based on the doped ZnO:Li_2_CO_3_, ZnO:Na_2_CO_3_, ZnO:K_2_CO_3_, and ZnO:Cs_2_CO_3_ EILs.

**EIL**	**V_on_^a^ (V)**	**Luminance^**b**^ (cd/m^**2**^)**	**CE^**b**^ (cd/A)**	**PE^**b**^ (lm/W)**
ZnO:5%Li_2_CO_3_	5.5	16,320	5.10	2.01
ZnO:5%Na_2_CO_3_	5.1	21,219	5.66	2.32
ZnO:5%K_2_CO_3_	4.8	22,651	6.04	2.61
ZnO:5%Cs_2_CO_3_	5.3	17,830	4.82	1.88

a *The driving voltage for the luminance of 1 cd/m^2^*.

b *The maximum values*.

In the IOLEDs, the transparency of the EIL in the visible wavelength region will greatly affect the output efficiency of devices, since the outgoing light has to pass through the EIL layer before going out. To investigate the M_2_CO_3_ doping effect on the transparency of the ZnO film, the transmittance spectra of the ZnO:M_2_CO_3_ EIL were characterized. As shown in Figure 2, all of the ZnO films exhibit high transparency in the visible wavelength range with transmittance values over 90%. It indicates that doping M_2_CO_3_ into the ZnO film has a minimal effect on the transmittance of the ZnO thin film, which is a promising property for making highly efficient IOLEDs.

Surface roughness of the ZnO EIL has been proven to have an influence on the device performance. The rough morphology of the EIL may cause partial short circuits and poor electrical contact in the thin flat devices, leading to dark spots in IOLEDs and thus poor device performance (Si et al., 2017). The morphology of the neat ZnO and doped ZnO:M_2_CO_3_ thin films were investigated by the tapping-mode AFM. As presented in Figure 3, the root-mean-square (RMS) roughness of the neat ZnO film is 0.67 nm, whereas, the RMS roughness of the doped ZnO film are 1.04, 0.93, 0.86, and 1.20 nm for ZnO:Li_2_CO_3_, ZnO:Na_2_CO_3_, ZnO:K_2_CO_3_, and ZnO:Cs_2_CO_3_ films, respectively. It can be seen that the morphologies of the ZnO:M_2_CO_3_ doped films are almost as smooth as that of the neat ZnO film, which is also beneficial to achieving high-performance IOLEDs.

To confirm that the M_2_CO_3_ exists in the doped ZnO films, we investigated the surface chemical composition of the neat ZnO and ZnO:M_2_CO_3_ using XPS analysis. Figure 4 shows Zn 2p_1/2_, Zn 2p_3/2_, O 1s, Li 1s, Na 1s, K 2p, and Cs 3d XPS spectra in comparison with those of the neat ZnO film. As shown in Figure 4A, the difference between Zn 2p_3/2_ and Zn 2p_1/2_ binding energies of both of the neat ZnO and doped ZnO:M_2_CO_3_ films is ~23 eV, which is in good agreement with the standard value of ~22.97 eV for Zn^2+^ (Hsien et al., 2008). When introducing the M_2_CO_3_ into the ZnO film, the Zn 2p_3/2_ and Zn 2p_1/2_ binding energies shift to slightly lower level, which may be attributed to the alkali metal cation doping effect (Chen et al., 2016). The O 1s XPS spectra (Figure 4B) of the neat ZnO film show a binding energy peak at 530.8 eV, which indicates that Zn-O exists in the neat ZnO film (Hsien et al., 2008). After the introduction of M_2_CO_3_ into the ZnO film, the O 1s binding energy shifts to slightly lower binding level (530.2 eV). In the same time, another binding energy band with a peak at ~532.3 eV appears, which indicates that the chemisorbed O_2_ molecules exists in the doped ZnO:M_2_CO_3_ film (Chen et al., 2016). As depicted in Figures 4C,D, the Li 1s peak, Na 1s peak, K 2p peak, and Cs 3d peak were clearly detected for the ZnO:Li_2_CO_3_ film, ZnO:Na_2_CO_3_ film, ZnO:K_2_CO_3_ film, and ZnO:Cs_2_CO_3_ film, respectively, which indicates that the alkali metal cation exists in the relative doped films.

To investigate the effect of M_2_CO_3_ doping on the device performance of IOLEDs, a series of green IOLEDs based on ZnO:M_2_CO_3_ EILs, with various doping concentrations (3, 5, and 10%) or various M_2_CO_3_ dopant (Li_2_CO_3_, Na_2_CO_3_, K_2_CO_3_, and Cs_2_CO_3_), were fabricated and characterized. Figure 5 represents the current density–luminance–voltage (J-L-V), the current efficiency and power efficiency characteristics for the IOLEDs, based on the neat ZnO or doped ZnO:K_2_CO_3_ EILs with various K_2_CO_3_ doping concentrations. The relative optoelectronic parameters are summarized in Table 1. As shown in Figure 5A and Table 1, the neat ZnO EIL based IOLED demonstrates a driving voltage of 5.2 V, a maximum luminescence of 15,430 cd/m^2^, a maximum current efficiency of 3.92 cd/A and a maximum power efficiency of 1.81 lm/W. By introducing the K_2_CO_3_ into the ZnO EIL, the devices show obviously enhanced device performance. When increasing the doping concentration of K_2_CO_3_, the operational voltage of the device would at first decrease and afterwards increase, whereas, the maximum luminescence, maximum current efficiency and maximum power efficiency show trends of rising first and then falling. As a result, the doped ZnO:5%K_2_CO_3_ EIL based IOLED possesses an optimal device performance with a decreased driving voltage of 4.8 V, and an enhanced maximum luminescence of 22,651 cd/m^2^, an increased maximum current efficiency of 6.04 cd/A and an improved maximum power efficiency of 2.61 lm/W. Compared with the neat ZnO based IOLED, the current efficiency of the doped ZnO:5%K_2_CO_3_ EIL based IOLED increased by 54%, which should be ascribed to the increased electron mobility and reduced barrier height for electron injection due to the doping of K^+^ ions, as discussed in a subsequent section.

Figure 6 demonstrates the J-V characteristics, L-V characteristics, current efficiency, and power efficiency characteristics of IOLEDs, based on doped ZnO:5%Li_2_CO_3_, ZnO:5%Na_2_CO_3_, ZnO:5%K_2_CO_3_, and ZnO:5%Cs_2_CO_3_ EILs, respectively. As shown in Figure 6 and Table 2, similar to the K_2_CO_3_ doping effect, introducing Li_2_CO_3_, Na_2_CO_3_, and Cs_2_CO_3_ into the ZnO EIL will obviously improve the maximum luminescence, maximum current efficiency, and maximum power efficiency of the IOLEDs compared with that of the neat ZnO based IOLED. As a familiar dopant in ZnO, the mechanism of Li ion doping leading to the enhancement of device performance has been well explained: the Li ion doped ZnO film possesses enhanced electron mobility and better band matching with adjoining layer (Chen et al., 2016). Herein, our results show that other alkali metal ions (Na, K, and Cs) can each also act as an efficient dopant in ZnO film for high efficiency OLED devices. Furthermore, the ZnO:5%K_2_CO_3_ EIL based IOLED displays the highest device performance. The difference in the device performance should be ascribed to the different electron injection properties of these devices caused by the different electron mobility and work function of the doped ZnO:M_2_CO_3_ films with various dopant, which may be attributed to them having a different ionic radius: Li (0.76 Å), Na (1.02 Å), K (1.38 Å), and Cs (1.67 Å) cation dopant (Chang et al., 2015). This leads to different interstitial or substitutional sites in the bulk ZnO thin films (Chang et al., 2015).

To compare the electron mobilities in the ZnO:M_2_CO_3_ EIL with various dopant, the electron-only devices, with a structure of ITO/ZnO:5%M_2_CO_3_ (10 nm)/Bphen (60 nm)/Liq (1 nm)/Al (120 nm) (Figure 7A), were also prepared and characterized. As depicted in Figure 7B, the electron-only device with ZnO:M_2_CO_3_ EIL exhibits much larger current density at the same voltage than that of the device with neat ZnO EIL. Moreover, the ZnO:K_2_CO_3_ EIL based electron-only device has the highest current density. This indicates that the electron mobilities of the ZnO:M_2_CO_3_ EIL are much higher than that of the neat ZnO EIL, and the electron mobility of the ZnO:K_2_CO_3_ EIL are highest in this series of ZnO:M_2_CO_3_ EILs. This observation is consistent with the device performance: the enhanced electron mobility will contribute to more efficient electron injection, and thus, higher device performance of the IOLEDs. Additionally, the effect of M_2_CO_3_ doping on the work function of the ZnO film was also studied. As shown in Table S1, the work function of the neat ZnO is 4.02 eV. Doping M_2_CO_3_ into the ZnO will effectively reduce the work function of the ZnO film. The ZnO:K_2_CO_3_ EIL has a much shallower work function of 3.61 eV. The obviously reduced work function of the ZnO:M_2_CO_3_ film has much better energy alignment with the lowest unoccupied molecule orbital (LUMO) level of the Bphen ETL in the IOLEDs. The reduced barrier height between EIL and ETL is beneficial for enhancing the charge injection efficiency, and thus, increasing the device performance of the ZnO:M_2_CO_3_ EIL based IOLEDs, especially in the ZnO:K_2_CO_3_ EIL case.

To further study the operational stability of ZnO:M_2_CO_3_ EIL in the IOLEDs, we chose the ZnO:K_2_CO_3_ EIL as an example to measure the operation lifetime of ZnO:M_2_CO_3_ EIL based IOLEDs. As shown in Figure S1, the operation lifetime declined to 50% after 25 h. The microscopic picture of the device shows that the dark strains occurred at the edge of the light-emitting areas. The unsatisfactory lifetime of the device may be attributed to the unstable property of the ZnO:K_2_CO_3_ film, possibly due to the presence of alkali metal K ion. As Takada et al. reported, the alkali metal ion may diffuse into the emitter layer of the OLED device under the action of the external voltage, which will cause quenching of the photoluminescence (Takada et al., 2017). In the same time, the air-stability of ZnO:M_2_CO_3_ EIL based IOLEDs were also studied. The result shows that the operation lifetime declined to 50% after 13 h (Figure S2). Figure S3 shows the image of the light-emitting areas of the ZnO:M_2_CO_3_ EIL based IOLED at a driving voltage of 9.0 V after 1, 3, and 24 h. The formation of dark spots and shrinkage from the edge of the light-emitting areas is clearly observed for the device without encapsulation kept in air over 3 h. The poor air-stability of the device should be also ascribed to the presence of alkali metal compounds in the IOLED devices. The alkali metal compounds have undesirable properties of strong affinity for moisture and reactivity with oxygen, which will significantly lead to the degradation of the OLED device (Sato et al., 2018).

## Conclusions

In summary, we have reported efficient IOLEDs employed solution-processed ZnO:M_2_CO_3_ as an EIL and Alq_3_ as an emitter layer. A series of M_2_CO_3_, including Li_2_CO_3_, Na_2_CO_3_, K_2_CO_3_, and Cs_2_CO_3_, were introduced into the ZnO EIL to enhance the electron injection efficiency of the IOLEDs. Our findings demonstrate that the doped ZnO:M_2_CO_3_ EIL-based IOLEDs possess obviously improved device performance, compared to the control neat ZnO EIL-based IOLEDs. An optimal current efficiency of 6.04 cd A^−1^ were obtained from the ZnO:K_2_CO_3_ EIL based IOLED, which is 54% improved than that of the neat ZnO EIL based device. The enhancement is ascribed to the increased electron mobility and reduced barrier height for more efficient electron injection. Our results indicate that the doped ZnO:M_2_CO_3_ EIL has promising potential for application in highly efficient solution-processed OLEDs.

## Author Contributions

GC and WZ: designed experiments; FL and ZL: carried out experiments; PZ: analyzed experimental results; GC, PZ, BW, and WZ: wrote the manuscript.

### Conflict of Interest Statement

The authors declare that the research was conducted in the absence of any commercial or financial relationships that could be construed as a potential conflict of interest.
