# Supplementary material for: Efficient Organic Light Emitting Diodes Using Solution-Processed Alkali Metal Carbonate Doped ZnO as Electron Injection Layer
Source: Front Chem. 2019 Apr 16;7:226. doi: 10.3389/fchem.2019.00226 (PMC6477122; doi:10.3389/fchem.2019.00226)
Supplement: Supplementary file 1 [file Data_Sheet_1.docx]

**Supplementary Information**

**Efficient Organic Light Emitting Diodes Using Solution-processed Alkali Metal Carbonate Doped ZnO as Electron Injection Layer**

Guo Chen^*^, Feiyang Liu, Zhitian Ling, Pengpeng Zhang, Bin Wei, Wenqing Zhu^*^

*Key Laboratory of Advanced Display and System Applications, Ministry of Education, Shanghai University, Yanchang Road 149, Shanghai 200072, China*

E-mail: [chenguo@shu.edu.cn](mailto:chenguo@shu.edu.cn); wqzhu@shu.edu.cn


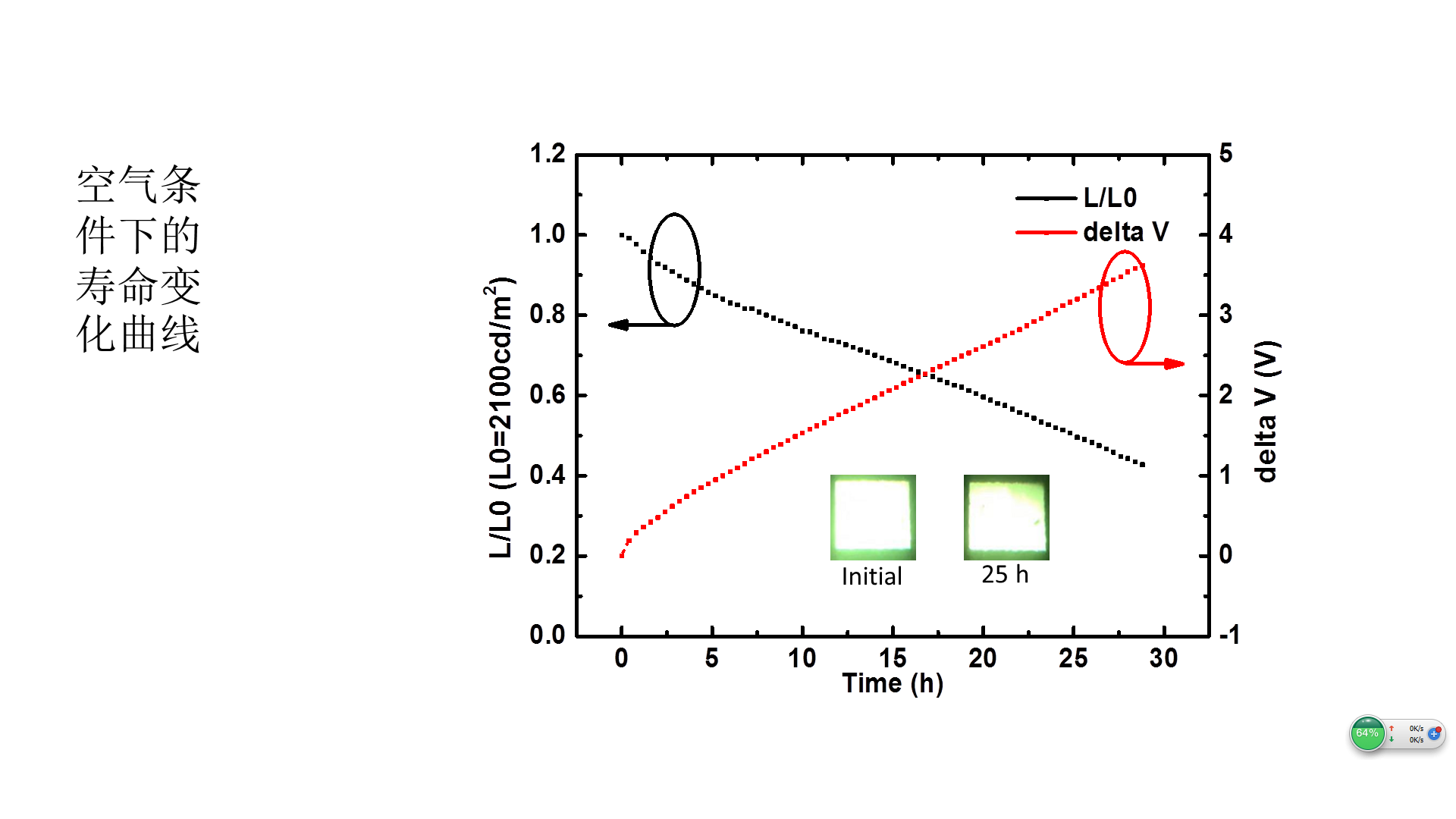


**Figure S1.** Time evolution of the normalized luminance, *L*, of ZnO:K_2_CO_3_ EIL based IOLEDs in a dry-nitrogen (<1 ppm H_2_O and O_2_) atmosphere and change in operating voltage *ΔV* (offset to zero) at the initial luminance of *L_0_* = 2100 cd m^-2^.





Figure S2. Time evolution of the normalized luminance, *L*, of ZnO:K_2_CO_3_ EIL based IOLEDs in air at the initial luminance of *L_0_* = 2100 cd m^-2^.


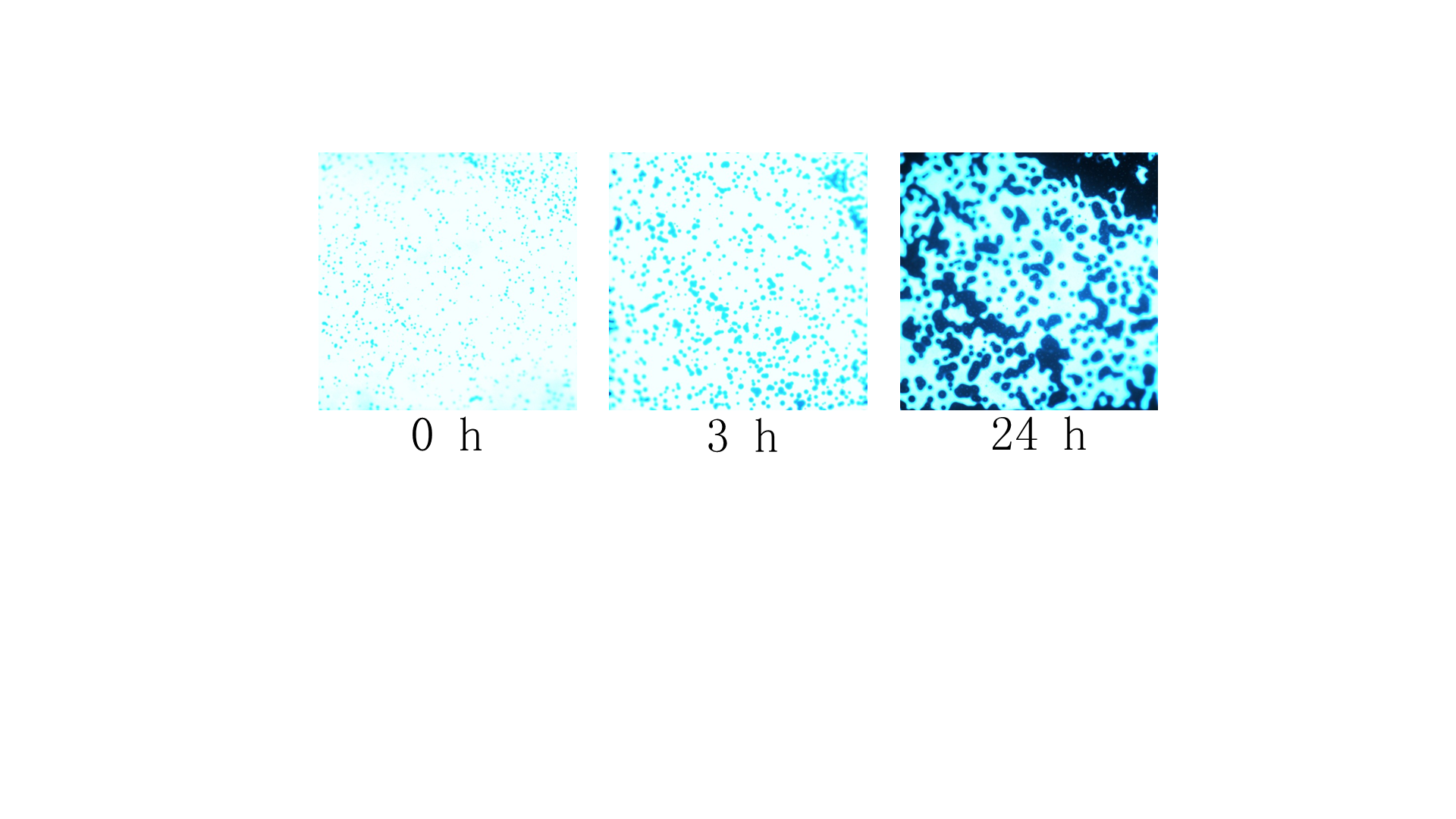


Figure S3. Image of light-emitting areas of ZnO:K_2_CO_3_ EIL based IOLEDs after air exposure for 0, 3, and 24 h. Here the device was keep in air atmosphere without encapsulation.

**Table S1** The work function of the neat ZnO and alkali metal carbonate doped ZnO films with various M_2_CO_3_ dopants.

| **Film** | ZnO | ZnO:5%Li_2_CO_3_ | ZnO:5%Na_2_CO_3_ | ZnO:5%K_2_CO_3_ | ZnO:5%Cs_2_CO_3_ |
| --- | --- | --- | --- | --- | --- |
| **Work Function (eV)** | 4.02 | 3.98 | 3.73 | 3.61 | 3.86 |
